# The Role of Chlorhexidine Gluconate (ChloraPrep™) in Reducing Surgical Site Infections After Ovarian Cancer Surgery

**DOI:** 10.3390/cancers18111726

**Published:** 2026-05-25

**Authors:** Mustafa Zelal Muallem, Andrea Miranda, Luigi Ferraro, Jalid Sehouli, Ahmed El-Balat

**Affiliations:** 1 Department of Gynecology with Center for Oncological Surgery, Charité-Universitätsmedizin Berlin, Corporate Member of Freie Universität Berlin and Humboldt-Universität zu Berlin, Augustenburger Platz 1, 13353 Berlin, Germany; andrea.miranda@charite.de (A.M.); luigi.ferraro@charite.de (L.F.); jalid.sehouli@charite.de (J.S.); 2 Department of Obstetrics and Gynecology, Sana Klinikum Offenbach, Starkenburgring 66, 63069 Offenbach am Main, Germany; dr.elbalat@gmail.com

**Keywords:** ovarian cancer, cytoreductive surgery, surgical site infection, chlorhexidine, cost-effectiveness, ERAS, infection prevention

## Abstract

**Background/Objectives**: Primary cytoreductive surgery for advanced ovarian cancer is associated with a high risk of postoperative complications, particularly surgical site infections, which may delay recovery and adjuvant treatment. This study aimed to evaluate the impact of a revised infection-prevention bundle, centered on chlorhexidine-alcohol skin antisepsis, on surgical site infection rates and cost-effectiveness. **Methods**: In this single-center cohort study, 636 patients undergoing primary cytoreductive surgery between January 2019 and December 2023 were included. A historical control group (*n* = 300) received povidone-iodine for intraoperative skin preparation, while a prospective intervention group (*n* = 336) received chlorhexidine gluconate 2% in 70% isopropyl alcohol. Both groups were managed within a standardized perioperative care pathway. Surgical site infections within 30 days were defined according to established criteria. Comparative and descriptive statistical analyses were performed. **Results**: Baseline clinicopathological characteristics were comparable between groups. The overall surgical site infection rate was significantly lower in the chlorhexidine-alcohol group compared with the povidone-iodine group (8.3% vs. 14.0%; *p* = 0.0226). The reduction was particularly evident in procedures lasting more than 180 min (9.5% vs. 17.1%; *p* = 0.0199), while no significant difference was observed in shorter procedures. Cost analysis demonstrated a net saving of approximately EUR 451 per procedure in the chlorhexidine group, driven by reduced infection-related costs and improved operating room efficiency. **Conclusions**: Baseline clinicopathological characteristics were comparable between groups. The overall surgical site infection rate was significantly lower in the chlorhexidine-alcohol group compared with the povidone-iodine group (8.3% vs. 14.0%; *p* = 0.0226). The reduction was particularly evident in procedures lasting more than 180 min (9.5% vs. 17.1%; *p* = 0.0199), while no significant difference was observed in shorter procedures. Cost analysis demonstrated a net savings of approximately EUR 451 per procedure in the chlorhexidine group, driven by reduced infection-related costs and improved operating room efficiency.

## Simple Summary

Surgery for advanced ovarian cancer is often complex and carries a high risk of postoperative infections, which can delay recovery and further treatment. Preventing these infections is essential to improve patient outcomes and reduce healthcare costs. In this study, we evaluated whether changing the skin disinfectant used before surgery could lower infection rates. Specifically, we compared a commonly used iodine-based solution with a chlorhexidine-alcohol preparation within a standardized care pathway. We found that the chlorhexidine-based antiseptic significantly reduced surgical site infections, especially in longer and more complex procedures. In addition, its use was associated with overall cost savings, despite slightly higher material costs. These findings suggest that optimizing perioperative infection prevention strategies can improve both clinical outcomes and resource utilization in ovarian cancer surgery, supporting the adoption of more effective antiseptic protocols in routine practice.

## 1. Introduction

The primary cytoreductive surgery (PCS) for advanced ovarian cancer (AOC) presents a high risk of severe postoperative complications due to the complexity of surgical treatment involving multi-visceral interventions [1,2]. These complications can lead to extended hospital stays, and patients face a delayed onset and/or a modified composition of their adjuvant treatment strategy, negatively affecting the prognosis and quality of life [3,4,5].

Among postoperative complications, surgical site infections (SSIs) represent a major clinical concern, as they are associated with increased morbidity, prolonged hospitalization, and higher healthcare costs [6,7,8,9].

Strategies to reduce SSIs have therefore become a key component of perioperative care, particularly within standardized pathways such as Enhanced Recovery After Surgery (ERAS) programs [10,11]. Enhanced Recovery After Surgery (ERAS) has long been implemented in our center and has contributed to improved perioperative outcomes in patients undergoing PCS for AOC [12]. One of the major complications of such surgery remains postoperative infection, especially SSI, occurring in 14.7% of our cases [1] and in 10–15% of cases in large observational and institutional cohorts [13,14].

In recent years, increasing attention has been directed toward optimizing intraoperative skin antisepsis as a modifiable factor in SSI prevention. Chlorhexidine-alcohol solutions have been shown in several randomized trials and meta-analyses to be more effective than povidone-iodine in reducing SSI rates across different surgical settings [15,16,17]. However, evidence remains heterogeneous, and data specifically addressing their role in complex oncologic procedures, such as primary cytoreductive surgery for advanced ovarian cancer, are limited.

Given the known elevated rates of SSI in patients undergoing PCS, as well as the importance of SSI reduction within value-based perioperative care pathways, our institution implemented a service-wide SSI reduction bundle for patients with AOC undergoing PCS [18].

The aim of this study was to evaluate the impact of this SSI-reduction bundle, with a particular focus on the introduction of chlorhexidine gluconate 2% in 70% isopropyl alcohol for intraoperative skin preparation, on the incidence of SSIs. In addition, we assessed the cost-effectiveness of this intervention in a real-world clinical setting. Compared with the previous protocol based on povidone-iodine, the revised bundle primarily introduced chlorhexidine-alcohol skin antisepsis to reduce the microbial burden at the surgical site. Additional measures, including the use of antibacterial sutures and standardized intraoperative practices, were implemented as part of the bundle.

We hypothesized that the implementation of this optimized infection-prevention strategy would lead to a clinically meaningful reduction in SSI rates, particularly in complex and prolonged procedures, while also providing economic benefits.

## 2. Materials and Methods

A total of 636 patients were enrolled in this study. In total, 336 prospective patients from April 2021 to December 2023 were part of the intervention group, where chlorhexidine gluconate 2% and isopropyl alcohol 70% (ChloraPrep™; Becton, Dickinson and Company, Franklin Lakes, NJ, USA) were used for intraoperative skin preparation, and 300 patients from January 2019 to April 2021 were part of the historic control group (retrospective), where povidone-iodine (Braunoderm™; B. Braun Medical AG, Carl-Braun-Straße 1 34212 Melsungen, Germany) was used as the intraoperative skin disinfection agent.

Preliminary results focused on the effectiveness of the disinfection agent in reducing postoperative morbidity. The main modification in the revised SSI-reduction bundle was the use of ChloraPrep™ for intraoperative skin disinfection instead of Braunoderm™. A second change was the routine use of antibacterial sutures (STRATAFIX™ Symmetric PDS™ Plus Knotless Tissue Control Device; Ethicon Inc., Johnson & Johnson, Somerville, NJ, USA) for fascial closure. Because it is difficult to distinguish the individual effects of these two major modifications on SSI outcomes, the observed results cannot be attributed solely to the use of ChloraPrep™. However, for simplicity and clarity, the historical cohort is referred to as the Braunoderm™ group, whereas the cohort treated under the revised SSI-reduction protocol is referred to as the ChloraPrep™ group.

The inclusion criteria comprised women with a primary diagnosis of ovarian, fallopian tube, or peritoneal cancer who were indicated for and underwent primary cytoreductive surgery, including all histological subtypes and all International Federation of Gynecology and Obstetrics (FIGO) stages. In addition, women with a first recurrence of ovarian, fallopian tube, or peritoneal cancer who were indicated for and underwent secondary cytoreductive surgery with the intention of complete tumor resection were also included.

The following exclusion criteria were defined: emergency surgical procedures like ileus operation, operations on infectious patients, or patients with immunosuppression, dementia or other conditions that impair comprehension, compliance or prognosis.

Intraoperative interventions included intravenous (IV) antibiotics per standard institutional guidelines and repeat dosing as needed; strict glycemic control; the use of separate sterile wound closing trays; a STRATAFIX™ Symmetric PDS™ Plus Knotless Tissue Control Device (Ethicon Inc., Johnson & Johnson, Somerville, NJ, USA), which was also treated with IRGACARE^®^ MP (triclosan) (BASF, Ludwigshafen, Germany); and donning new sterile gloves at the time of fascial closure. Triclosan is known to inhibit bacterial colonization of sutures that are commonly associated with surgical site infections and to create zones of inhibition [19].

Standard institutional guidelines for the administration of intraoperative IV antibiotics were devised with the input of the infectious disease department and included the administration of the first dose of prophylactic antibiotics within 30–60 min before the initial surgical incision to ensure adequate serum and tissue levels [20].

Redosing was required if the duration of the procedure exceeded two half-lives of the antimicrobial agent (in 3 h) or if there was excessive blood loss during the procedure (defined as >1500 mL). Standard prophylaxis was with one dose of cefuroxime 1.5 g IV + metronidazole 500 mg IV, both administered 30–60 min before incision. Patients with a severe penicillin allergy were alternatively administered one dose of 600 mg clindamycin IV plus one dose of 3 mg/kg gentamicin IV. The placement of intraperitoneal or subcutaneous drains was left to the discretion of the attending gynecologic surgeon and was not specified within the SSI reduction bundle.

The incidence of SSIs and wound dehiscence was analyzed within 30 days of surgery. SSIs were defined as per Centers for Disease Control and Prevention (CDC) guidelines and categorized by depth and tissue space involvement [21].

This study involves human participants and was approved by the ethical institutional committee board of Charité Medical University EA1/287/20 (as part of KORE-OVAR-INNOVATION [18]). Participants gave informed consent to participate in the study before taking part.

The statistical analysis was performed at the Charité Medical University Berlin. All analyses were performed by IBM SPSS Statistics 21.0 (SPSS, Chicago, IL, USA). Continuous variables were summarized using medians and ranges, while categorical variables were reported as frequencies and percentages. Differences between the Braunoderm and ChloraPrep™ groups were assessed using the Mann–Whitney U test for continuous variables and the chi-square test or Fisher’s exact test, as appropriate, for categorical variables.

The primary endpoint was the occurrence of surgical site infection (SSI). Crude odds ratios (ORs) with 95% confidence intervals (95% CIs) were calculated using univariable logistic regression analyses.

The present analysis was based on retrospectively aggregated institutional data in the historical control group (Braunoderm). Patient-level datasets containing all covariates simultaneously—including age, operative time, bowel resection, residual disease, FIGO stage, ascites, and SSI status for each individual patient—were not available in a format permitting reliable multivariable logistic regression modeling. Consequently, only univariable comparisons between groups could be performed. Therefore, adjusted odds ratios (aORs) could not be reliably calculated, and residual confounding cannot be excluded.

Operative time was analyzed either as a continuous variable or categorized according to clinically relevant thresholds (>180 min versus ≤180 min). Residual disease was categorized as no residual disease, residual disease < 10 mm, and residual disease ≥ 10 mm. FIGO stage was entered as stage II, III, or IV, and ascites as no ascites, <500 mL, or ≥500 mL.

Model fit and multicollinearity diagnostics were assessed prior to interpretation of the final model. A two-sided *p*-value < 0.05 was considered statistically significant.

## 3. Results

### 3.1. Patient Characteristics

A total of 636 patients were included, with 300 in the Braunoderm group and 336 in the ChloraPrep™ group. The two cohorts were comparable with respect to baseline clinicopathological characteristics. Patient characteristics are summarized in Table 1. The median age at first diagnosis was 63 years (range 25–87) in the Braunoderm group and 62 years (range 23–86) in the ChloraPrep™ group. Similarly, median preoperative serum CA-125 levels did not differ substantially (380 U/mL vs. 367 U/mL).

The distribution of FIGO stages was comparable between the two groups (FIGO II: 6.3% vs. 6.5%; FIGO III: 75.7% vs. 76.8%; FIGO IV: 18.0% vs. 16.7%; *p* = 0.9047). No significant differences were observed regarding tumor grading (grades 1–2: 8.7% vs. 9.8%; grade 3: 82.7% vs. 83.9%; *p* = 0.4699). Histological subtypes were also similarly distributed, with serous papillary carcinoma being predominant (92.7% vs. 94.6%; *p* = 0.8507).

Assessment of intraoperative ascite volume revealed comparable findings across both groups (no ascites: 29.3% vs. 28.0%; <500 mL: 39.7% vs. 37.2%; >500 mL: 31.0% vs. 34.8%; *p* = 0.5904). Overall, no statistically significant differences were detected in any of the examined baseline variables, indicating that the two treatment cohorts were well balanced.

### 3.2. Surgical Outcomes and Procedure Characteristics

Surgical characteristics and surgical site infection outcomes are summarized in Table 2. No significant differences were observed between the two antiseptic groups regarding residual tumor status (no residual disease vs. <10 mm vs. ≥10 mm; χ^2^(2) = 0.045, *p* = 0.978) or the frequency of bowel resections (57.3% in the Braunoderm group vs. 58.0% in the ChloraPrep™ group; χ^2^ = 0.03, *p* = 0.858; Fisher’s exact *p* = 0.873). The overall rate of surgical SSI was lower in the ChloraPrep™ group (8.3%) compared with the Braunoderm group (14.0%), representing a statistically significant difference (χ^2^ = 5.196, *p* = 0.0226; Fisher’s exact *p* = 0.0304; OR ≈ 0.56, _95%_CI: 0.33–0.93). Therefore, the odds of surgical site infection were about 44% lower in the ChloraPrep™ group compared with the Braunoderm group. In procedures lasting <180 min, SSI rates did not differ significantly between groups (5.8% vs. 8.4%; χ^2^ = 0.558, *p* = 0.455; Fisher’s exact *p* = 0.594). However, in surgeries exceeding 180 min, ChloraPrep™ was associated with a significantly lower SSI rate (9.5% vs. 17.1%; χ^2^ = 5.424, *p* = 0.0199; Fisher’s exact *p* = 0.0288; OR ≈ 0.51, _95%_CI: 0.28–0.91). Therefore, the odds of SSI were about 49% lower with ChloraPrep™ in surgeries exceeding 180 min. Operative duration was reported as medians (Braunoderm 315 min [range 66–654] vs. ChloraPrep™ 306 min [Range: 38–662]); a formal statistical comparison was not possible without individual-level data.

### 3.3. Cost-Effectiveness Analysis

A cost-effectiveness analysis was conducted comparing 100 patients undergoing preoperative skin antisepsis with ChloraPrep™ versus 100 patients treated with Braunoderm™. The evaluation incorporated three economic components:

- Operating room (OR) time expenditure associated with skin preparation;
- Antiseptic acquisition costs;
- Costs attributable to SSI.

SSI rates were derived from our cohort, demonstrating a reduction from 14% in the Braunoderm group to 8.3% in the ChloraPrep™ group, corresponding to six prevented infections per 100 procedures. The economic impact of SSIs was estimated using the lowest published cost per SSI from Arefian et al. (2016) [22], amounting to EUR 5879 per SSI-event, without adjusting for inflation.

This 6% reduction in SSIs by using ChloraPrep™ in comparison with Braunoderm™ translates to EUR 35,274 saved per 100 procedures (equivalent to EUR 352.74 saved per procedure).

Moreover, ChloraPrep™ enabled a reduction in preoperative disinfection time of 10 min per case. Applying the OR time cost estimate of EUR 10.80 per OR minute reported by Rougereau et al. [23], this results in an additional EUR 108 saved per procedure through improved workflow efficiency. We must state here that primary cytoreductive surgery for advanced ovarian cancer represents one of the most resource-intensive procedures in gynecologic oncology, with substantially higher OR costs than standard surgical interventions. While average OR minute costs in German hospitals are commonly reported in the range of EUR 5–15 [24,25,26], the complexity, duration, and staffing needs of maximal-effort cytoreduction substantially elevate these values. Considering multi-disciplinary involvement, high consumption of surgical materials, prolonged anesthesia, and extensive use of specialized equipment, a realistic estimate for this procedure is approximately EUR 20–40 per OR minute. It is worth mentioning that a universal “cost per minute of surgery” in Germany is difficult to define because it depends on many factors (type of surgery, staff, equipment, consumables, operating room, anesthesia, turnover times, etc.). But we chose the most conservative published data about the estimated OR costs per minute to simplify our financial analysis.

**Figure 1:**
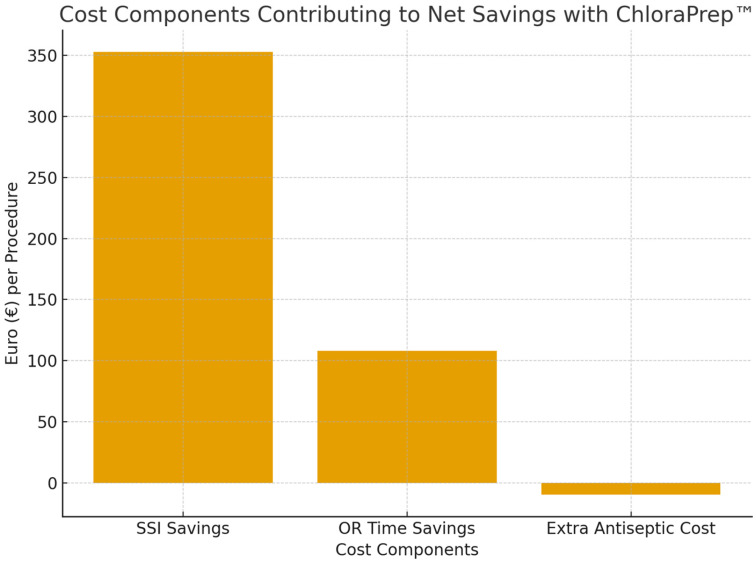
Cost components contributing to net savings per procedure with ChloraPrep™. Net savings result from reduced surgical site infections and operating room time and are only marginally impacted by additional antiseptic material costs.

Material costs differed between groups: antiseptic use for Braunoderm™ was approximately EUR 1 per procedure (200 mL), whereas ChloraPrep™ required one 10.5 mL and one 26 mL applicator, totaling EUR 10.47 per procedure, i.e., an incremental antiseptic cost of EUR 9.47 when using ChloraPrep™. Taking all components into account, ChloraPrep™ generated EUR 460.74 in combined savings per procedure (infection-related and time-related) while adding EUR 9.47 in antiseptic material cost. Thus, the net gain associated with ChloraPrep™ use was EUR 451.27 saved per procedure, demonstrating clear dominance and overall cost-effectiveness compared with Braunoderm™ in this cohort. As shown in Figure 1, net savings are primarily driven by reductions in infection-related costs and operating room time, while higher antiseptic material costs account for only a small additional cost component.

## 4. Discussion

In this study, we evaluated the impact of a revised SSI-reduction bundle—centering on the replacement of povidone-iodine with chlorhexidine gluconate 2% in 70% isopropyl alcohol (ChloraPrep™)—on postoperative outcomes in women undergoing primary cytoreductive surgery for advanced ovarian cancer. Our analysis demonstrated that the introduction of ChloraPrep™ was associated with a lower overall rate of surgical site infections, with an exploratory subgroup analysis showing a statistically significant reduction in procedures lasting >180 min. The two cohorts were broadly comparable with respect to major clinicopathologic baseline variables and indicators of surgical complexity, which may reduce the likelihood that the observed differences were solely related to baseline imbalances. Nevertheless, given the retrospective historical-control design and the absence of multivariable adjustment, residual confounding and temporal bias cannot be excluded. Furthermore, formal interaction testing was not performed; therefore, the subgroup findings should be interpreted cautiously and considered hypothesis-generating.

The reduction in SSIs from 14.0% in the Braunoderm™ group to 8.3% in the ChloraPrep™ group represents a clinically meaningful improvement, given the high morbidity associated with postoperative infections following cytoreductive surgery. These findings are consistent with results from Memorial Sloan Kettering Cancer Center, where implementation of a multimodal SSI-reduction bundle—including preoperative washing with 4% chlorhexidine gluconate—significantly lowered SSI rates in patients with gynecologic malignancies undergoing colon surgery. In that cohort, the incidence of SSI decreased from 37% to 12%. Notably, their overall SSI rate was substantially higher than that observed in our population, a difference that likely reflects their inclusion of only patients undergoing bowel resection, whereas bowel surgery was performed in approximately 57.7% of patients in our study [27].

Evidence from the broader surgical literature further supports our findings. A systematic review of 27 studies including 17,735 patients reported that only 2.0–2.5% chlorhexidine in alcohol (RR 0.75, 95% CI 0.61–0.92) and 1.5% olanexidine solutions (RR 0.49, 95% CI 0.26–0.92) significantly reduced SSI rates compared with aqueous iodine-based preparations [17].

Due to the retrospective historical-control design and lack of complete patient-level covariate data suitable for regression modeling, multivariable adjustment for potential confounders could not be performed. Therefore, residual confounding cannot be excluded. However, several mechanisms may explain the superior performance of chlorhexidine-alcohol solutions. Compared with povidone-iodine, chlorhexidine offers a more rapid onset of antimicrobial activity, sustained residual effect, and broad-spectrum efficacy against Gram-positive and Gram-negative bacteria. The enhanced effect observed in procedures lasting longer than 180 min is particularly noteworthy. Prolonged operative time is a well-established risk factor for SSI, related to cumulative bacterial exposure, tissue desiccation, and increased opportunities for sterility breaches [28,29]. The persistent antimicrobial activity of chlorhexidine—especially its resistance to inactivation by blood and body fluids—may therefore yield greater benefit in prolonged, high-complexity procedures such as maximal cytoreductive surgery for advanced ovarian cancer.

In addition to the observed clinical outcomes, our analysis suggests a potential economic benefit associated with the adoption of ChloraPrep™. Although the acquisition costs of the antiseptic were higher, the lower SSI incidence together with the shorter preoperative skin-preparation time was associated with estimated net savings of approximately EUR 451 per procedure. These findings are consistent with previous economic analyses in other surgical fields demonstrating that reductions in SSI rates may translate to meaningful cost savings due to the substantial costs associated with the management of postoperative infections.

Furthermore, our cost calculations were intentionally conservative, as they were based on the lowest published SSI-related cost estimates and operating-room time valuations, which may underestimate the actual resource utilization associated with extensive cytoreductive surgery. Consequently, the economic impact observed in routine clinical practice may differ and could potentially be greater than estimated in the present analysis. However, given the retrospective design and assumptions underlying the cost model, these findings should be interpreted cautiously and considered exploratory.

Although the study included a large number of consecutive patients treated under a standardized ERAS pathway, several limitations merit consideration. First, the retrospective historical-control design introduces the potential for temporal bias. Although operative strategies, surgical staffing, perioperative antibiotic protocols, and institutional ERAS pathways remained largely unchanged during the study period, unmeasured temporal improvements in perioperative management, surgical workflow, team experience, or compliance with perioperative care measures may have influenced SSI rates independently of the antiseptic regimen. Consequently, a causal relationship between ChloraPrep™ use and reduced SSI risk cannot be established with certainty. In addition, although measured baseline characteristics were comparable between groups, residual confounding from unmeasured variables cannot be excluded. Finally, the SSI-reduction bundle included multiple components; therefore, the isolated effect of ChloraPrep™ cannot be determined definitively, although the transition to chlorhexidine-alcohol represented the principal antiseptic modification during the study period. An additional limitation of this study is that the primary focus was on short-term postoperative infectious complications within 30 days, and long-term oncologic or survival-related outcomes were not evaluated. These results were presented partially in a previous study [2] and will be published and discussed comprehensively in a separate paper.

Despite these limitations, the strengths of this study include its large sample size, real-world clinical applicability, and the focus on a homogeneous population undergoing one of the most complex procedures in gynecologic oncology. Our findings reinforce the growing body of surgical literature demonstrating the superiority of chlorhexidine-alcohol solutions in preventing SSIs and provide novel evidence supporting their benefit in primary cytoreductive surgery for advanced ovarian cancer.

## 5. Conclusions

The implementation of ChloraPrep™ as part of an optimized SSI-reduction bundle significantly decreased postoperative infectious complications and demonstrated strong cost-effectiveness in patients undergoing primary cytoreductive surgery for ovarian cancer. These results support the adoption of chlorhexidine-alcohol as the preferred intraoperative skin antiseptic in gynecologic oncology and highlight the importance of evidence-based perioperative infection-prevention strategies to improve both patient outcomes and healthcare resource utilization.

Future multicenter prospective studies with patient-level datasets and predefined multivariable analyses are warranted to confirm the independent effect of ChloraPrep™ on surgical site infection rates and to validate the reproducibility of our findings across broader clinical settings.

## Figures and Tables

**Table 1:** Patient characteristics.

Characteristics	Braunoderm™ *n* = 300 (%)	ChloraPrep™ *n* = 336 (%)	All Patients *n* = 636 (%)	*p*-Value
**Age at first diagnosis** [years]	Median 63 (25–87)	Median 62 (23–86)	Median 62 (23–87)	
**CA-125**	380 U/mL	367 U/mL	348 U/mL	
**FIGO classification**				
II	19 (6.3%)	22 (6.5%)	41 (6.4%)	0.9047
III	227 (75.7%)	258 (76.8%)	485 (76.3%)	
IV	54 (18%)	56 (16.7%)	110 (17.3%)	
**Grading**				
1–2	26 (8.7%)	33 (9.8%)	59 (9.3%)	0.4699
3	248 (82.7%)	282 (83.9%)	530 (83.3%)	
Not defined	26 (8.7%)	21 (6.3%)	47 (7.4%)	
**Histology**				
Serous papillary	278 (92.7%)	318 (94.6%)	596 (93.7%)	0.8507
Mucinous	9 (3%)	6 (1.8%)	15 (2.4%)	
Endometrioid	5 (1.7%)	4 (1.2%)	9 (1.4%)	
Clear cell	4 (1.3%)	4 (1.2%)	8 (1.3%)	
Mixed	4 (1.3%)	4 (1.2%)	8 (1.3%)	
**Ascites**				
No ascites	88 (29.3%)	94 (28%)	182 (28.6%)	0.5904
<500 mL	119 (39.7%)	125 (37.2%)	244 (38.4%)	
>500 mL	93 (31%)	117 (34.8%)	210 (33%)	

**Table 2:** Surgery characteristics and surgical site infection.

Characteristics	Braunoderm *n* = 300 (%)	ChloraPrep™ *n* = 336 (%)	All Patients *n* = 636 (%)	*p*-Value
**Residual tumor**				
No residual	216 (72%)	241 (71.7%)	457 (71.9%)	0.978
<10 mm	59 (19.7%)	68 (20.2%)	127 (20%)	
≥10 mm	25 (8.3%)	27 (8%)	52 (8.2%)	
**Bowel resection**	172 (57.3%)	195 (58%)	367 (57.7%)	0.858
**Operation duration** [minutes]	Median 315 (66–654)	Median 306 (38–662)	Median 311 (38–662)	
**Surgical site infections**	42 (14%)	28 (8.3%)	70 (11%)	0.0226
**Surgical site infections according to the operating time**				
<180 min	8.4% (9/107)	5.8% (6/104)	7.1% (15/211)	0.558
>180 min	17% (33/193)	9.5% (22/232)	12.9% (55/425)	0.0199

## Data Availability

The data presented in this study are available on reasonable request from the corresponding author. The data are not publicly available due to privacy restrictions.

## Abbreviations

The following abbreviations are used in this manuscript:

AOC	advanced ovarian cancer
CDC	Centers for Disease Control and Prevention
ERAS	Enhanced Recovery After Surgery
FIGO	International Federation of Gynecology and Obstetrics
IV	intravenous
OR	operating room
PCS	primary cytoreductive surgery
SSI	surgical site infection
